# Development of a non-invasive exhaled breath test for the diagnosis of head and neck cancer

**DOI:** 10.1038/s41416-020-01051-9

**Published:** 2020-09-09

**Authors:** Nuwan Dharmawardana, Thomas Goddard, Charmaine Woods, David I. Watson, Eng H. Ooi, Roger Yazbeck

**Affiliations:** 1 Department of Otorhinolaryngology-Head and Neck Surgery, Flinders Medical Centre, Bedford Park, Australia; 2 Discipline of Surgery, College of Medicine and Public Health, Flinders University, Bedford Park, Australia; 3 Department of Respiratory and Sleep Medicine, Women’s and Children’s Hospital, North Adelaide, Australia

**Keywords:** Diagnostic markers, Head and neck cancer, Head and neck cancer, Diagnostic markers, Translational research

## Abstract

**Background:**

Improving the ability to identify early-stage head and neck squamous cell carcinoma (HNSCC) can improve treatment outcomes and patient morbidity. We sought to determine the diagnostic accuracy of breath analysis as a non-invasive test for detecting HNSCC.

**Methods:**

Standardised breath samples were collected from 181 patients suspected of HNSCC prior to any treatment. A selected ion flow-tube mass spectrometer was used to analyse breath for volatile organic compounds. Diagnosis was confirmed by histopathology. A binomial logistic regression model was used to differentiate breath profiles between cancer and control (benign disease) patients based on mass spectrometry derived variables.

**Results:**

In all, 66% of participants had early-stage primary tumours (T1 and T2) and 58% had regional node metastasis. The optimised logistic regression model using three variables had a sensitivity and specificity of 80% and 86%, respectively, with an AUC for ROC curve of 0.821 (95%CI 0.625–1.0) in the testing cohort.

**Conclusions:**

Breath analysis for non-invasive diagnosis of HNSCC appears to be practical and accurate. Future studies should be conducted in a primary care setting to determine the applicability of breath analysis for early identification of HNSCC.

## Background

Mucosal head and neck squamous cell carcinomas (HNSCC) arise from the nasopharynx, oropharynx, hypopharynx, larynx and the oral cavity. Classical risk factors for HNSCC include tobacco and alcohol consumption. However, more recently, a surge in human papilloma virus (HPV)-associated oropharyngeal cancers have been reported in the US,^1^ Canada^2^ and UK.^3^ These cancers are also affecting a much younger population without classical risk factors for HNSCC.^4^ Worldwide, HNSCC accounts for 6% of all cancers and up to 2% of cancer-related deaths.^5^ Current therapies are effective at treating early-stage disease, with limited morbidity; however, late-stage presentations are common, and often associated with poor prognosis and high treatment-related morbidity.^6^ Therefore, methods of early detection are needed to improve the treatment outcomes of HNSCC patients.

As primary care assessment of the complete upper aerodigestive tract is technically limited, tertiary referral for investigation of suspected head and neck cancer is required, and largely driven by symptoms.^7^ A critical analysis of such symptoms against a large cohort of patients (*n* = 4715) in the UK, found the most sensitive (45.4%) indicator to be an unexplained neck lump present for more than 3 weeks, but this only had a specificity of 79.6%.^7^ The presence of a cranial neuropathy provided the best positive predictive value (66.7%). However, a combined symptom model only produced a maximum area under the curve (AUC) of 0.78.^7^ When a patient develops a neck mass or cranial neuropathies, the stage of their HNSCC is likely to be advanced.^4^ Therefore, earlier diagnosis is needed, and biomarker studies for earlier cancer detection have potential to enhance and improve current symptom-based HNSCC detection methods.^8^

In the last decade, volatile organic compounds (VOC) have gained interest as biomarkers for cancer detection. VOCs are organic molecules with a high vapour pressure at ambient temperature and can be measured using mass spectrometers and gas detectors. Humans emit a range of VOCs from various body fluids including exhaled breath, with a recent meta-analysis identifying more than 250 VOCs with the potential to detect human cancer using breath analysis.^8^ Twenty four of these compounds from four studies were able to detect HNSCC.^9–12^ Despite these promising findings, these studies did not analyse the entire breath profile (complete mass spectra) of the patient, potentially ‘missing’ novel breath biomarkers for HNSCC. The objective of this multicentre study was to determine whether the breath profile can be used to discriminate patients with vs. without HNSCC.

## Methods

### Ethical approval and consent

Ethical approval (HREC reference number HREC/16/SAC/70) was obtained from Southern Adelaide Local Health Network Human Ethics Committee with site-specific approvals for Flinders Medical Centre and Royal Adelaide Hospital, Adelaide, South Australia. Informed consent was obtained from all participants prior to sample collection. Local and international guidelines were followed as per the Declaration of Helsinki for research involving human participants.

### Patients

Patients referred to the Flinders Medical Centre and Royal Adelaide Hospital Head and Neck clinics, following assessment by an experienced Otolaryngologist with a clinical suspicion of HNSCC, were recruited for this study. These patients all had a histological diagnosis of SCC arising in the mucosa of the oral cavity, oropharynx or larynx, based on biopsy of the primary cancer under general anaesthesia or metastatic sites. A control group consisted of healthy adult patients who presented for pan-endoscopy with upper aerodigestive symptoms and clinical suspicion of HNSCC, but subsequently had a normal pan-endoscopy examination or histologically benign biopsy results.

Exclusion criteria included a histological diagnosis of high-grade dysplasia, patients with other concurrent malignancy or history of malignancy, patients with head and neck cutaneous malignancies, patients aged <18 years, and patients with an active inflammatory condition or infection. Patients with SCC in neck lymph nodes with no identifiable primary mucosal tumour (Unknown Primary HNSCC) were also excluded.

Patients with an oropharyngeal primary lesion also had an immunohistochemical characterisation of p16 as an indirect surrogate (prognostic) marker for human papilloma virus (HPV). Patients with HNSCC were staged using the 8th Edition of the American Joint Committee on Cancer (AJCC) Cancer Staging Manual.^13^ Co-morbidities and medication intake were recorded and categorised based on American Society of Anaesthesiologists (ASA) grade (Table 1).

**Table 1:** Comparison of demographics, comorbidities and medications between cancer and control patient groups.

Variable	Control group No. (%)	Cancer group No. (%)	*P* value
Count	50 (50)	50 (50)	
Age—Years, median (Range)	56 (31–86)	58 (33–88)	0.104^b^
Sex			<0.001^a^*
Female	25 (50)	7 (14)	
Male	25 (50)	43 (86)	
Smoking			0.311^a^
Never smoked	19 (38)	11 (22)	
Ex-smoker	15 (30)	22 (44)	
Current smoker	16 (32)	17 (34)	
Smoking Pack Years (Range)	23.6 (0–104)	24.0 (0–198)	
Alcohol			0.264^a^
No alcohol intake	18 (36)	11 (22)	
Intake ≤2 days per week	19 (38)	17 (34)	
Intake ≥3 days per week	13 (26)	22 (44)	
BMI	28.5 (17.9–45.4)	25.6 (17.3–38.2)	0.002^b^*
BMI Class^d^			0.118^a^
Under Weight	1 (2)	3 (6)	
Normal Weight	11 (22)	20 (40)	
Pre-Obesity	18 (36)	17 (34)	
Obesity: Class I	14 (28)	8 (16)	
Class II	2 (4)	2 (4)	
Class III	4 (8)	0	
ASA grade			0.328^a^
1	34 (68)	33 (66)	
2	12 (24)	17 (34)	
3	4 (8)	0	
Comorbidities			0.623^a^
Ischaemic heart disease	3 (6)	5 (1)	
Chronic respiratory disease	7 (14)	8 (16)	
Chronic renal disease	0	0	
Chronic liver disease	1 (2)	1 (2)	
Diabetes	6 (12)	6 (12)	
Medications^c^			0.126^a^
Anti-reflux	20 (40)	9 (18)	
Anti-hypertensives	12 (24)	18 (36)	
Antibiotics	0	0	
Anti-platelet/coagulant	2 (4)	5 (1)	
Tooth Brushing^e^			0.015^a^*
Yes	41 (84)	31 (62)	
No	8 (16)	19 (38)	

*Statistical significance (*p*  <  0.05).

^a^Chi-square test.

^b^Mann-Whitney-U test.

^c^Medications taken in the last seven days are reported.

^d^Body mass index (BMI) class reported as per World Health Organization classification.

^e^Tooth brushing in the morning of breath sample collection.

### Breath collection

Patients who were planned for the elective procedure were contacted the day before sample collection and to reduce any contamination of the breath sample they were instructed not to wear perfume or deodorant sprays, not to brush their teeth, not to smoke and not to use any mouth wash on the morning of sample collection. Patients mainly adhered to all above instructions, except 73% of patients reported brushing their teeth prior to sample collection. The median time between tooth brushing and breath collection was 3 h (Range 2–6 h).^14^ Patients fasted overnight for a minimum of 6 h as per anaesthetic requirements for the planned surgical procedures. All exhaled breath samples were collected prospectively in the peri-operative setting prior to any surgical procedures or anaesthetic administration.

On arrival to the hospital, patients were asked to rest in a bed (in a four to six bay ward) for at least fifteen minutes prior to sample collection. During the rest period, a structured patient history was collected using a questionnaire prior to sample collection. This included smoking status, smoking pack years, alcohol intake (days per week), comorbidities, height, weight, fasting time, tooth brushing, mouth wash use and chewing gum use. They were then asked to take a deep breath in through the nose, followed by a single continuous forced exhalation through the mouth (while pinching their nostrils closed) into a sealed 3 litre FlexFoil® PLUS® bag (SKC Ltd, Pennsylvania, USA) resulting in a mixed alveolar gas sample (mixture of alveolar air and respiratory dead space air).^15^ While the capacity of samples bags was 3 litres, the exhaled breath volume varied among patients (not measured). A room air sample was collected into another FlexFoil® PLUS® bag immediately after the breath sample collection for comparison and quality assurance. Samples were then transported in a temperature stable bag (Esky®, Coleman Brands, New South Wales, Australia) maintained at 37 °C (Deltaphase® isothermal pads, Braintree Scientific Inc, Massachusetts, USA) to the laboratory for immediate analysis. All FlexFoil® PLUS® bags were preconditioned by flushing with 99% nitrogen gas five times prior to breath sample collection.

### Mass spectrometry

On arrival at the lab, samples were stored in a dedicated 37 °C incubator and were analysed within three hours of collection by selected ion flow-tube mass spectrometry (SIFT-MS; Voice 200^®^, Syft^®^ Technologies, Christchurch, New Zealand). The SIFT-MS was calibrated twice daily (morning and afternoon) using a standard gas mix (1,2,3,4-Tetrafluoro benzene, benzene, ethylene, isobutane, octafluorotoluene, p xylene, perfluorobenzene, toluene and nitrogen – Scotty® specialty gases, Pennsylvania, USA).

For sample analysis, the septum of the SKC^®^ FlexFoil^®^ PLUS^®^ bag was pierced with a non-coring needle and attached directly to the SIFT-MS ‘breath-head’ (Voice 200^®^, Syft^®^ Technologies, Christchurch, New Zealand). All samples (including room air) were scanned using the full mass scan mode (15–250 m/z) with three reagent ions (H_3_O^+^, NO^+^, O_2_^+^) for 30 cycles. Approximately one litre of the gas sample was used for SIFT-MS analysis; however, SIFT-MS analysis sensitivity is not volume dependent. The instrument corrected intensities for all mass to charge ratios were extracted from the instrument generated files. In all, 15 mL of the exhaled breath samples were also analysed by Isotope Ratio Mass-Spectrometry (Sercon®, Crewe, United Kingdom) for carbon dioxide quantification. Samples found to have <3% carbon dioxide in breath were excluded from further analysis, as this would be a less than expected carbon dioxide level from a mixed alveolar breath sample.^16^

### Data analysis

The sample size required for statistical modelling was calculated based on the prevalence of disease and fixed type I error with at least 80% power. The point prevalence of HNSCC in our dataset was artificially set to 50% by balancing the cancer and control groups. Sample size calculation tables published by Bujang et al.^17^ were used to estimate a minimum total sample size of 62, with a minimum of 31 patients with HNSCC, to test for 70% sensitivity and 70% specificity with 80% power.

All statistical analyses were performed using IBM^®^ SPSS^®^ (Version 26. Chicago, United States, with R^©^ version 3.5 - extensions). Normality of data distribution was tested using the Kolmogorov–Smirnov test. Fixed patient factors were compared between cancer and control groups using Mann–Whitney-U (MWU) test for continuous fixed patient factors (age, smoking pack years and body mass index [BMI]) and Chi-squared test for categorical fixed patient factors (gender, smoking status, alcohol intake, BMI class, ASA, comorbidities, medications and tooth brushing status) (Table 1). The associations between categorical patient factors and SIFT-MS derived variables were assessed using the Mann–Whitney U test (with Bonferroni correction). Spearman’s rho test was used to determine associations between continuous patient factors with SIFT-MS derived variables. Statistically significant association was considered if *p* < 0.05.

**Fig. 1:**
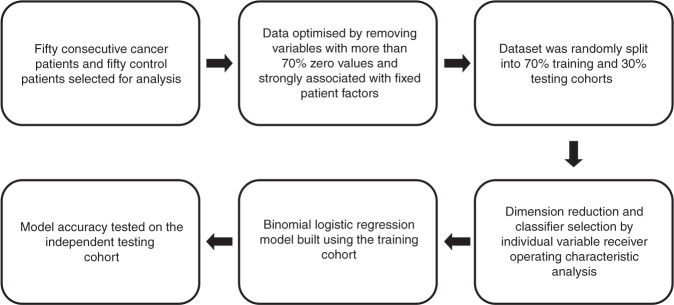
Data analysis pipeline for case selection, data optimisation, training/testing data split, dimension reduction, model building and model testing.

A zero-value analysis was conducted, where variables with more than 70% zero value readings were excluded as it was difficult to ascertain if the zero reading was due to limit of detection of the instrument or it was a true zero reading for that sample. Masses known to conflict with reagent ions (reported by the instrument manufacturer) were also removed from further analysis. Variables that indicated a significant association to fixed patient factors were also excluded. Following the data optimisation process, 440 variables were available for predictive analysis. The dataset was then randomly split into 70% (*n* = 76) training and 30% (*n* = 24) testing datasets using the SPSS ‘Select Cases’ function (Supplementary Table 1). The data analysis workflow is summarised in Fig. 1.

### Dimension reduction and predictor selection

Using the training dataset, a ROC analysis with the AUC were calculated and ranked for individual SIFT MS derived variables. Two further methods were employed for predictor selection. First, the top 10 variables for each of the reagent ions were extracted (Supplementary Table 2). Second, only the statistically significantly different variables (mix of all three reagent ions) from baseline AUC of 0.5 were selected (Supplementary Table 3).

### Logistic regression model

Patient gender and BMI were significantly different between control and cancer groups (Table 1). Therefore, predictive performance of these fixed factors was individually assessed in the logistic regression model (Supplementary Table 4). A series of logistic regression models were generated using the training dataset based on the two sets of variables selected above, to achieve a ROC AUC above 0.8 with sensitivity and specificity above 70% in the testing dataset, while minimising the number of variables used (Supplementary Tables 5 and 6). Confidence intervals reported here were adjusted based on a 1000 sample bootstrapping procedure for improved certainty. Variables included in the final models were re-tested on associated patient factors to confirm lack of fixed factor dependent bias (Supplementary Table 7).

## Results

A total of 181 patients were recruited. 74 patients were excluded due to previous history of cancer (*n* = 23), high grade dysplasia (*n* = 7), unknown primary HNSCC (*n* = 3), active infection or poor carbon dioxide concentration in the breath sample (*n* = 41). Consecutive control (*n* = 50) and cancer (*n* = 50) patients were selected from the remaining 107 patients for further analysis to maintain pre-test probability at 0.5 (50%-artificial point prevalence). Breath sampling and processing did not result in any adverse events. Total time to collect a patient breath sample after instruction and demonstration was approximately one minute.

Control group patients had a normal upper aerodigestive tract examination, or a benign biopsy result following histological analysis. The HNSCC group were predominantly male (*p* = 0.001) and had a lower BMI than the control group (*p* = 0.002). There was no significant difference in age, smoking status, smoking pack years and alcohol intake between groups (*p* > 0.05). The majority of patients in the control group had an ASA grade of 2 or lower with no incapacitating comorbidities. However, four control patients were classified as ASA 3 due to morbid obesity (Table 1). There was a significant difference between tooth brushing status between cancer and control patient groups (*p* = 0.015). Cancer-specific factors including head and neck subsite and tumour stage are reported in Table 2. HNSCC patients largely comprised of oropharyngeal SCC (46%), then oral cavity SCC (32%), and laryngeal SCC (30%). Nineteen (83%) of the oropharyngeal SCCs were p16 positive. Regional node metastasis was present in 58% of patients. The majority (68%) of the HNSCC patients were early T-stage (T1 or T2) (Table 2).

**Table 2:** Description of cancer specific factors.

Cancer related factor	Patients, No. (%)
Head and neck subsite	
Oral Cavity	16 (32)
Oropharynx	23 (46)
p16 positive	19
p16 negative	4
Larynx	15 (30)
Clinical T stage	
1	16 (32)
2	17 (34)
3	7 (14)
4	10 (20)
Neck node status	
Negative	21 (42)
Positive	29 (58)
Overall prognostic stage	
I	18 (36)
II	11 (22)
III	9 (18)
IV	12 (24)

### Predictive modelling

**Fig. 2:**
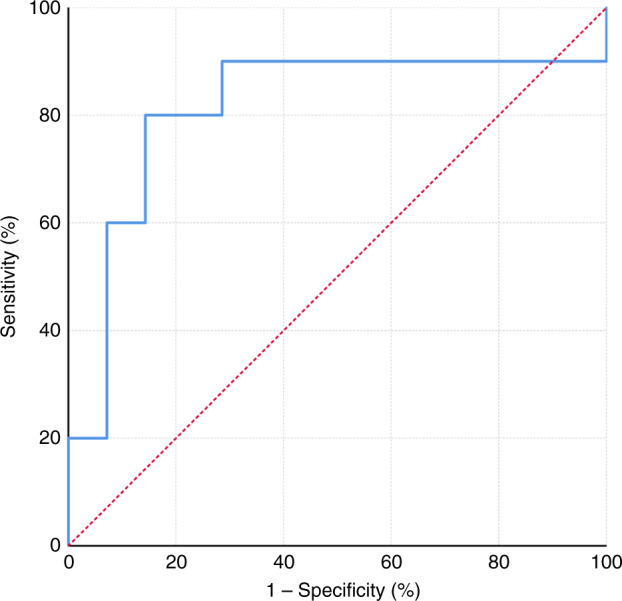
Model 1—Receiver operating curve of the testing cohort based on logistic regression model using the two variables from distinct reagent ions.

**Fig. 3:**
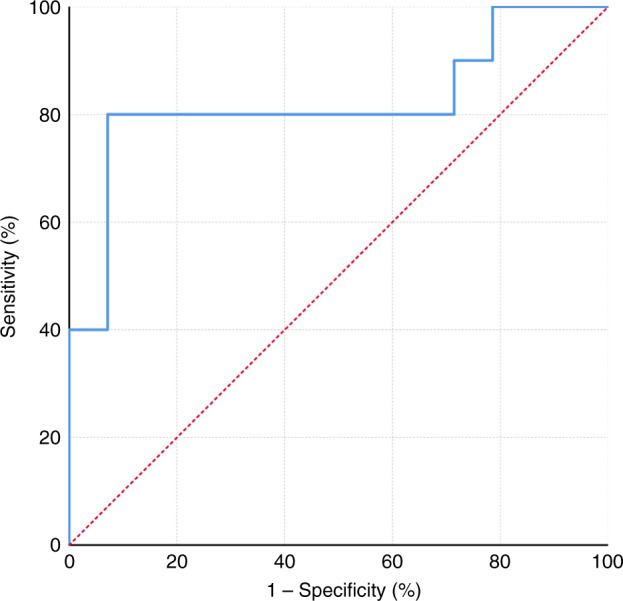
Model 2—Receiver operating curve of the testing cohort based on logistic regression model with one variable from each reagent ion (three variables in total).

Binomial logistic regression models generated on mass spectrometry derived variables from exhaled breath were able to classify patients to cancer vs. control groups. The accuracy of this classification was maintained when tested on the independent cohort with an AUC up to 0.9 with 15 variables (Supplementary Table 5). However, maintaining the model sensitivity and specificity and reducing over-fitting of the data resulted in an optimal model with two variables (Model 1) with an overall classification accuracy of 83% (AUC of 0.814 95% CI 0.611–1.000), sensitivity of 80% and specificity of 86% (Fig. 2). This model used the variables R30P147 and R19P49. These variables belong to two distinct reagent ions and adding a third variable (Model 2) from another reagent ion (R32P135) marginally improves the overall model (AUC 0.821 95% CI 0.625–1.000, Fig. 3).

Gender specific classification analysis of Model 1 using the testing cohort indicated a 79 and 90% overall accuracy for the male and female cohorts, respectively. The variables contained in Model 1 (R30P147 and R19P49) were used to generate new models with gender as the dependent variable (Supplementary Table 8). These variables as individual classifiers and in combination performed poorly at predicting gender with a maximum training set AUC of 0.591 (95% CI 0.458–0.723) and testing set AUC of 0.5 (95% CI 0261–0.739). There was no linear relationship (based on linear regression) between BMI and the variables used in Model 1 or Model 2 (Supplementary Table 7). Data exploration with principal components analysis with the variables used for Model 1 and Model 2 also did not classify the patients based on gender, BMI or tooth brushing status (Supplementary Fig. 1).

The independently curated Syft® compound library was interrogated using LabSyft® (version 1.5.1, Syft®, Christchurch, New Zealand) software to identify the VOCs contributing to the product mass ions included in modelling described above. VOCs with poor branching ratios (<0.2), poor reaction rates (<1.0 e^−11^), and isomers with the same primary chemistry were excluded. Two of the ten compounds that corresponded to the underlying chemistry of the SIFT-MS derived variables have been previously described as predictive of lung and oral cavity cancer (Table 3).^18,19^

**Table 3:** Volatiles organic compounds corresponding to product mass ions of interest.

Variable	VOCs	Product Formulae	Chemistry	Carcinogenicity/toxicity
R19P49				
Reagent ion H_3_O^+^	Formaldehyde*^18^	H_2_CO.H^+^.H_2_O	Secondary	Known carcinogen
Product m/z 49	Methyl mercaptan*^19^	CH_4_S.H^+^	Primary	Acute toxicity
	Sevoflurane	CH_2_FO^+^	Primary	Irritant
R30P147				
Reagent ion NO^+^	Benzyl cyanide	C_8_H_7_N.NO^+^	Primary	Acute toxicity
Product m/z 147	Coumarin	C_9_H_6_O_2_^+^.H^+^	Secondary	Suspected carcinogen
	Cuminal	C_10_H_11_O^+^	Primary	Irritant
R32P135				
Reagent ion O_2_^+^	2-aminoacetophenone	C_8_H_9_NO^+^	Primary	Non-toxic
Product m/z 135	Amphetamine	C_9_H_13_N^+^	Primary	Acute toxicity
	Benzothiazole	C_7_H_5_NS^+^	Primary	Acute toxicity
	Carvacrol	C_9_H_11_O^+^	Primary	Irritant

Indicated carcinogenicity and toxicity is based on details from pubchem.ncbi.nlm.nih.gov. Previously reported predictive nature of cancer was indicated with * and relevant citation.

## Discussion

This study describes a novel breath test for the detection of HNSCC, with a sensitivity of 80% and specificity of 86% in the optimal model. The robustness of this breath test was demonstrated by the consistently high performance noted in the independent testing cohort. The predictive ability of this test is significantly higher than that reported for clinical symptoms and examination alone,^7^ suggesting that raw mass spectra breath analysis for HNSCC has the potential to improve current clinical practice. Additionally, this study presents a novel method of analysing SIFT-MS derived raw data.

Patients with suspected head and neck cancer often present to a primary care setting with non-specific upper aerodigestive symptoms; however, complete examination by the general practitioner is restricted by technical limitations. Whilst the oral cavity, part of the oropharynx and the external neck can be examined thoroughly by general practitioners, the nasopharynx, base of tongue and the larynx can only be examined in specialist clinics with the use of flexible endoscopes. In addition, early-stage HNSCC are unlikely to show overt macroscopic clinical signs and may not be visible in cross-sectional imaging. Therefore, when a patient presents to a primary care physician with upper aerodigestive symptoms without a clear risk profile associated with HNSCC, instead of considering antibiotics or deciding to watch and wait, an exhaled breath test might be considered. Given the high sensitivity and specificity of our breath test, we hypothesis that a positive result could trigger an urgent specialist referral for further evaluation. A negative result could support a short period of observation, or medical treatment with further investigations instigated should symptoms persist. The intention of such a breath test would not be to replace clinical expertise or diagnostic procedures such as a pan-endoscopy and biopsy under general anaesthesia. Instead, a breath test might provide an triaging system to risk stratify patients and potentially alleviate some pressure from healthcare resources.^20^

An acknowledged limitation of our study was that gender and BMI were significantly different between comparison groups (Table 1). From an epidemiological perspective, HNSCC predominantly affects the male population^21^ and weight loss is a well-documented hallmark sign of malignancy and a marker of poor prognosis.^22^ We used univariate statistical comparisons prior to classifier selection to ensure the variables used in model generation were not associated with gender or BMI. The variables selected for these models did not classify patients based on gender and BMI, indicating the chosen classifiers are not solely dependent on these patient factors (Supplementary Table 7 and Supplementary Fig. 1). Furthermore, exhaled breath compounds reported to be gender^23^ and BMI^24^ specific were not associated with the SIFT-MS derived variables included in our final model. Future studies are indicated using larger cohorts to validate models based on gender or BMI specific groups.

There was a significant disparity regards to tooth brushing status prior to sampling between comparison groups (Table 1). In this study the association between tooth brushing status and classifiers were addressed using univariate statistical comparison and excluded prior to model generation. Vadhwana et al. have reported the effects of various oral cleansing methods on sampled breath VOCs that were sampled immediately after oral cleansing, finding differences based on the type of cleanser, compound of interest and the time between said cleansing and breath sampling.^14^ Exhaled menthone, decane, dodecane and p-cresol concentrations increased while ammonia, butanoic acid and dimethyl disulphide concentrations decreased with no reported changes to aldehydes, alcohol and some hydrocarbon levels after toothbrushing.^14^ In our study, participants that had brushed their teeth had done so at least three hours prior to breath sampling, minimising any potential effects on the breath VOCs reported. Furthermore, there were no associations between the compounds prone to variate based on toothbrushing and the variables selected for model generation in this study (Table 3). Nonetheless, future validation studies in larger cohorts should seek to determine an optimum and consistent oral cleansing method immediately prior to breath collection.

SIFT-MS allows for full mass scan of a sample as well as calculating concentrations of individual VOCs and has been comprehensively described by Spanel et al.^25^. By utilising the SIFT-MS product-ion mass-spectra, the complete breath profile of the participant can be interrogated, removing any bias associated with VOC concentration calculations.^25^ This method potentially allows for cross-platform comparison of datasets, facilitating capacity for multi-centre studies. Schumtzhard et al. have reported the analysis of raw mass spectra of breath samples for HNSCC using proton transfer reaction mass spectrometry,^26^ comparing the H_3_O^+^ product masses between HNSCC (*n* = 22) and control patients (benign, high-risk and post-treatment). They found 42 masses that were significantly different between groups but did not report sensitivity or specificity for direct comparison. Although, none of them (or the corresponding VOCs identified using the Syft® library) were reported in the recent meta-analysis by Hanna et al. as predictive of cancer.^8^ We identified Formaldehyde and Methyl mercaptan as potential VOCs related to cancer with a product mass of 49 based on reagent H_3_O^+^ (Table 3). Exhaled formaldehyde has been reported to be predictive of primary lung cancer^18^ and it is thought to be from methylotrophic bacteria present in the aerodigestive tract.^27,28^ Microbial dysbiosis has been reported in cancer, which could contribute to a distinctive VOC profile. Similarly, methyl mercaptan is a volatile sulphur compound detected in the human oral cavity and is widely implicated in periodontal disease and halitosis.^19^ Clinically, patients with oral cavity malignancies present with concurrent halitosis. While poor oral hygiene or halitosis is not a direct risk factor for cancer, objective measurement of halitosis with volatile sulphur compounds such as methyl mercaptan may assist in risk stratifying patients with poor oral hygiene.

Four prior studies using breath analysis to detect HNSCC have reported sensitivities ranging from 77% to 100% and specificities ranging from 76% to 92%.^9–12^ The total sample size in these studies ranged between 41 and 62 with unequal groups.^9–12^ While these results are promising, the statistical power based on sample size alone is low. In comparison, our study had a larger sample size, with 50 cancer and 50 control patients, providing 80% statistical power for determining sensitivity and specificity above 70%, much larger sample would be required for independent validation.^17^ However, only 30% of HNSCC patients in our current study were T1 and T2 stage with no lymph node metastasis, thus limiting statistical power to determine the accuracy of this model for identifying early-stage HNSCC. Hence, prior to recommending this breath test for routine clinical use, a large-scale diagnostic accuracy study should be performed in the primary care setting. Given the relatively low incidence of HNSCC, in order to adequately power (>80%) a primary care study, more than 5000 participants would be required. The large-scale multi-site trial (PAN Study, ClinicalTrials.gov Identifier: NCT03756597) to investigate breath analysis for the detection of early cancer in patients across the UK represents an example of the study design necessary to validate this new technology.^29^

Clinically, the most convenient use of a breath test would be as a point of care device, providing an immediate result for clinical decision making. Advanced smart gas sensors (electronic noses) are routinely used in defence and environmental protection agencies for environmental toxic gas detection.^30^ Various nanomaterial-based sensors have also been developed for specific VOC detection properties for the development of point of care devices.^31^ These arrays of very sensitive gas detectors can profile complex gas samples, such as human breath using computerised pattern recognition to discriminate cancer from healthy patients.^9,10^ Therefore, the product ion masses identified in this study could potentially be used to calibrate these devices to specifically detect HNSCC, thereby presenting a pathway for clinical translation.

## Conclusion

This is the largest human study using an exhaled breath test to detect HNSCC, and it identified a panel of VOCs with a sensitivity of 80% and a specificity of 86%. The next stage is to determine the diagnostic accuracy for early-stage HNSCC compared to non-cancer controls in a primary care setting. Cross-instrument validation of these findings are also important for direct comparison to other breath analysis techniques. Nonetheless, this breath test shows promise as an adjunct for improving the detection of HNSCC.

## Supplementary information

Supplementary Material

## Data Availability

Modelling data are provided in this manuscript as well as in supplementary tables. Deidentified raw data is available upon request.
