# Supplementary material for: Development of a non-invasive exhaled breath test for the diagnosis of head and neck cancer
Source: Br J Cancer. 2020 Sep 9;123(12):1775–81. doi: 10.1038/s41416-020-01051-9 (PMC7722848; doi:10.1038/s41416-020-01051-9)
Supplement: Supplementary file 1 — Supplementary Material [file 41416_2020_1051_MOESM1_ESM.pdf]

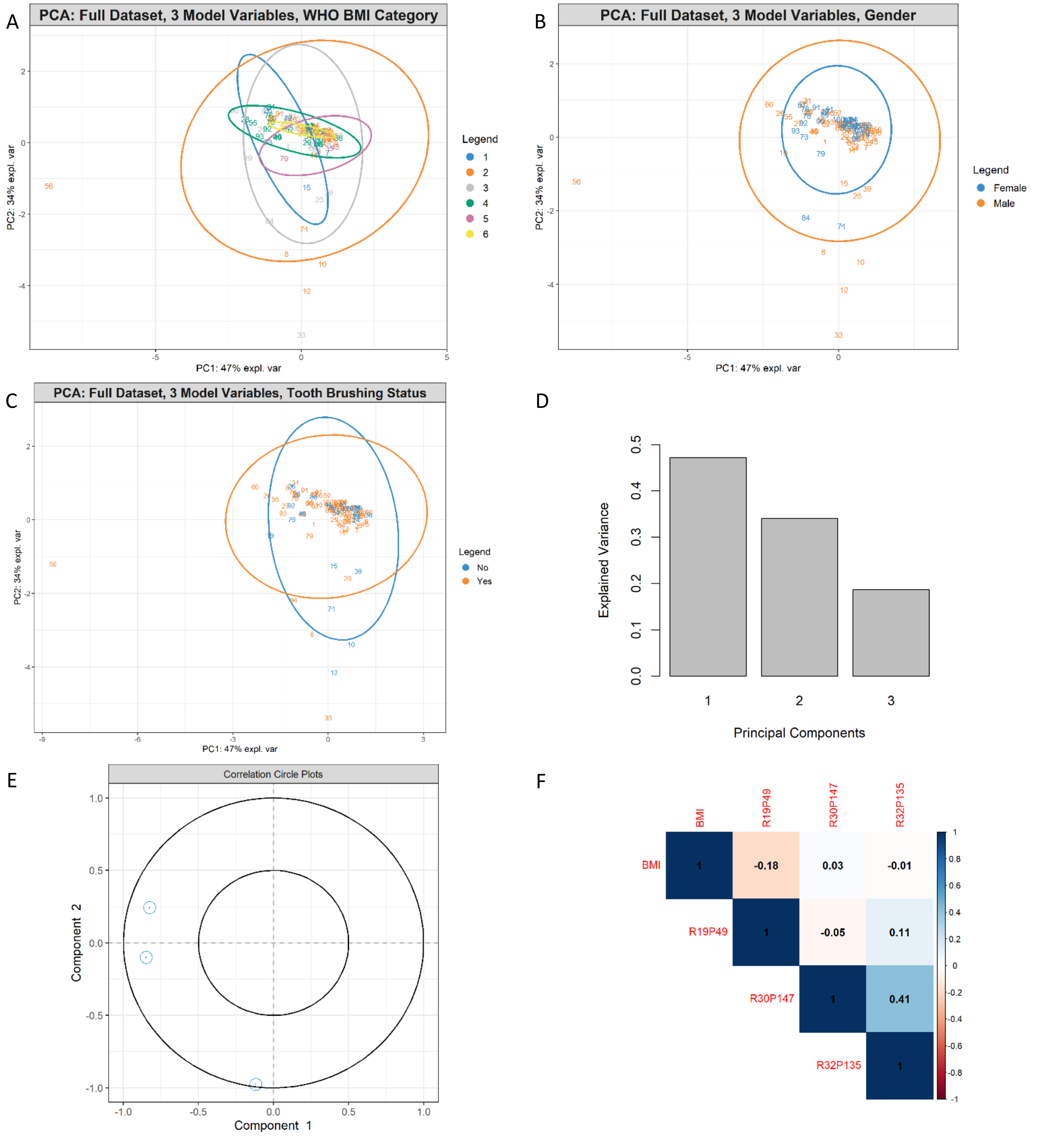

Supplementary Figure 1. Unsupervised sample classification and spearman correlation of the complete modelling dataset based on variables selected for the final logistic regression model. A, PCA classification labelled based on WHO BMI category. B, PCA classification labelled based on gender. C, PCA classification labelled based on tooth brushing status. D, PCA total variance explained by the first three components. E, Spearman correlation of variables contained in the first two principal components. F, Spearman correlation between BMI and the three variables used for the final logistic regression model. PCA, principal components analysis. BMI, body mass index. WHO, World Health Organisation.

Supplementary Table 1. Comparison of significantly different patient factors between training and testing patient cohorts

Variable	**Training**		**P Value**	**Testing**		**P Value**
	Control	Cancer		Control	Cancer	
n	36	40		14	10	
Sex			0.005 [a] *			0.008 [a] *
Female	16	6		9	1	
Male	20	34		5	9	
BMI	30.22	26.51	0.005 [b] *	27.5	23.44	0.026 [b] *
Brush Teeth			0.130 [a]			0.022 [a] *
Yes	29	26		1	5	
No	7	14		12	5	

*a, Chi-square test. b, Mann-Whitney-U test. *, statistical significance ($p < 0.05$).*

Supplementary Table 2. Variables selected and ranked based on ROC-AUC per reagent

Rank	Variable Name		
	R19 (H_3O^+)	R30 (NO^+)	R32 ($O2^+$)
1	**R19P49**	**R30P147**	**R32P135**
2	R19P125	R30P143	R32P109
3	R19P30	R30P187	R32P82
4	R19P189	R30P211	R32P138
5	R19P59	R30P240	R32P31
6	R19P18	R30P64	R32P47
7	R19P81	R30P188	R32P184
8	R19P48	R30P80	R32P40
9	R19P133	R30P95	R32P103
10	R19P77	R30P159	R32P95

Supplementary Table 3. Variables selected based on overall ROC-AUC

Rank	Variable Name
1	**R30P147**
2	**R19P49**
3	R30P143
4	R30P187
5	R19P125
6	R30P211
7	R30P240
8	R32P135
9	R19P30
10	R32P109
11	R30P64
12	R30P188
13	R30P80
14	R32P82
15	R30P95
16	R30P159
17	R32P138
18	R32P31

Supplementary Table 4. Prediction based on gender and BMI

	Train	n=76			Test	n=24		
Variables	Sen	Spe	AUC	95% CI	Sen	Spe	AUC	95% CI
G	85	44.4	0.647	0.521-0.773	90	64.3	0.771	0.577-0.966
BMI	41	80.6	0.654	0.530-0.777	60	64.3	0.693	0.482-0.904
G + BMI	87.2	47.2	0.725	0.610-0.841	90	57.1	0.764	0.575-0.954

G, gender. BMI, body mass index.

Supplementary Table 5. Variables selected based on AUC ranking per reagent ion

	Train	n=76				Test	n=24			
Variables	Sensitivity	Specificity	AUC	Lower 95% CI	Upper 95% CI	Sensitivity	Specificity	AUC	Lower 95% CI	Upper 95% CI
30	100.00%	100.00%	1	1	1	40.00%	64.30%	0.529	0.284	0.773
27	100.00%	100.00%	1	1	1	60.00%	64.30%	0.579	0.332	0.826
24	95.00%	88.90%	0.978	0.953	1	70.00%	71.40%	0.621	0.387	0.856
21	82.50%	80.60%	0.929	0.876	0.983	70.00%	50.00%	0.493	0.243	0.743
18	80.00%	69.40%	0.874	0.799	0.949	80.00%	57.10%	0.814	0.625	1
15	75.00%	72.20%	0.862	0.783	0.94	90.00%	85.70%	0.9	0.748	1
12	67.50%	69.40%	0.815	0.722	0.908	70.00%	78.60%	0.779	0.587	0.97
9	75.00%	77.80%	0.806	0.705	0.908	80.00%	71.40%	0.786	0.585	0.987
6	75.00%	69.40%	0.774	0.668	0.881	40.00%	64.30%	0.586	0.342	0.83
3	**75.00%**	**66.70%**	**0.754**	**0.645**	**0.863**	**80.00%**	**85.70%**	**0.821**	**0.625**	**1**

Supplementary Table 6. Variables selected based on overall AUC ranking

	Train	n=76				Test	n=24			
Variables	Sensitivity	Specificity	AUC	Lower 95% CI	Upper 95% CI	Sensitivity	Specificity	AUC	Lower 95% CI	Upper 95% CI
10	72.50%	75.00%	0.817	0.723	0.912	80.00%	71.40%	0.707	0.482	0.932
9	75.00%	72.20%	0.808	0.711	0.904	80.00%	71.40%	0.729	0.507	0.95
8	70.00%	63.90%	0.795	0.697	0.893	70.00%	64.30%	0.671	0.441	0.902
7	72.50%	63.90%	0.797	0.699	0.894	60.00%	64.30%	0.657	0.42	0.894
6	77.50%	63.90%	0.79	0.687	0.892	50.00%	71.40%	0.629	0.384	0.874
5	72.50%	69.40%	0.785	0.683	0.888	50.00%	71.40%	0.664	0.422	0.907
4	70.00%	66.70%	0.752	0.643	0.861	80.00%	64.30%	0.857	0.688	1
3	72.50%	66.70%	0.737	0.624	0.851	70.00%	78.60%	0.779	0.571	0.986
2	**72.50%**	**66.70%**	**0.747**	**0.633**	**0.86**	**80.00%**	**85.70%**	**0.814**	**0.611**	**1**
1	80.00%	36.10%	0.702	0.583	0.822	80.00%	71.40%	0.725	0.485	0.965

Supplementary Table 7. Linear regression between included model variables and BMI on testing cohort

Variables	**Adjusted R^2**	**F statistic**	**P value**
R32P135 + R30P147+R19P49	0.041	0.696	0.565
R32P135 + R19P49	0.014	0.838	0.446
R30P147 + R19P49	0.016	0.82	0.454

R30P147 + R32P135	0.03	0.67	0.523
R19P49	0.016	0.643	0.431
R30P147	0.01	1.242	0.277
R32P135	0.024	0.455	0.507

Supplementary Table 8. Predict gender (male) using final model variables (R30P147 and R19P49)

	**Train**	**n=76**			**Test**	**n=24**		
Variables	Sen	Spe	AUC	95% CI	Sen	Spe	AUC	95% CI
R30P147	100%	0%	0.582	0.449-0.715	100%	0%	0.5	0.261-0.739
R19P49	100%	0%	0.581	0.439-0.724	100%	0%	0.5	0.261-0.739
R19P49 + R30P147	100%	0%	0.591	0.458-0.723	100%	0%	0.5	0.261-0.739
